# Long noncoding RNA ANRIL indicates a poor prognosis of gastric cancer and promotes tumor growth by epigenetically silencing of miR-99a/miR-449a

**DOI:** 10.18632/oncotarget.1902

**Published:** 2014-04-16

**Authors:** Er-bao Zhang, Rong Kong, Dan-dan Yin, Liang-hui You, Ming Sun, Liang Han, Tong-peng Xu, Rui Xia, Jin-song Yang, Wei De, Jin fei Chen

**Affiliations:** ^1^ Department of Biochemistry and Molecular Biology, Nanjing Medical University, Nanjing, Jiangsu, PR China;; ^2^ Central laboratory, the Second Affiliated Hospital of Southeast University, Nanjing, Jiangsu, PR China;; ^3^ Department of Oncology, Xuzhou Central Hospital, Affiliated Xuzhou Hospital, College of Medicine, Southeast University, Xuzhou, Jiangsu, PR China;; ^4^ Department of Oncology, First Affiliated Hospital of Nanjing Medical University, Nanjing, Jiangsu, PR China;; ^5^ Department of Oncology, Nanjing First Hospital, Nanjing Medical University, Nanjing, jiangsu, PR China

**Keywords:** ANRIL, PRC2, miR-99a/miR-449a, proliferation, gastric cancer

## Abstract

Long noncoding RNAs are involved in diseases including cancer. Here, we reported that ANRIL (CDKN2B-AS1), a 3.8-kb long noncoding RNA, recruiting and binding to PRC2, was generally upregulated in human gastric cancer (GC) tissues. In a cohort of 120 GC patients, the higher expression of ANRIL was significantly correlated with a higher TNM stage (P=0.041) and tumor size (P=0.001). Multivariate analyses revealed that ANRIL expression served as an independent predictor for overall survival (P=0.036). Further experiments revealed that ANRIL knockdown significantly repressed the proliferation both in *vitro* and in *vivo*. We also showed that E2F1 could induce ANRIL and ANRIL-mediated growth promotion is in part due to epigenetic repression of miR-99a/miR-449a in *Trans* (controlling the targets—mTOR and CDK6/E2F1 pathway) by binding to PRC2, thus forming a positive feedback loop, continuing to promote GC cell proliferation. To our knowledge, this is the first report showed that the role of ANRIL in the progression of GC and ANRIL could crosstalk with microRNAs in epigenetic level. Our results suggest that ANRIL, as a growth regulator, may serve as a candidate prognostic biomarker and target for new therapies in human gastric cancer.

## INTRODUCTION

Gastric cancer (GC) is the second leading cause of cancer death, and is the most common gastrointestinal malignancy in East Asia, Eastern Europe, and parts of Central and South America[1]. Gastric cancer is diagnosed at advanced stage accompanied by malignant proliferation in most patients and the prognosis for advanced stage patients is still very poor[2]. Although many oncogenes and tumor suppressors have been identified as key players underlying tumorigenesis of GC, however, almost no commonly-accepted biomarkers have been established to facilitate the comprehensive management of patients, for example prognostic prediction. Therefore, the identifications of the new biomarkers and therapeutic targets for GC and a detailed understanding of the molecular mechanisms underlying gastric carcinogenesis will supply an arm for improving diagnosis and treatment of human GC.With the development of whole-genome sequencing technology, it was determined that less than 2% of the mammalian genome is in protein-encoded regions and the remainder is in noncoding RNAs (ncRNAs)[3]. Among them are long noncoding RNAs (lncRNAs), which are more than 200 nt in length and unable to be translated into proteins. Recently, many lncRNAs are known to play important roles in cellular development, differentiation, and many other biological processes [4-9]. The dysregulation of lncRNAs have also been shown in various types of cancer including GC [10-13], suggesting that they may display a large regulatory component of the eukaryotic genome.

The molecular mechanisms of lncRNAs are complex, they have been shown to regulate gene expression at various levels, including chromatin modification, transcription and post-transcriptional processing [14, 15]. For example, lncRNA HOTAIR is overexpressed in breast tumors and could promote metastasis by the interaction with the PRC2 (Polycomb Repressive Complex 2) [10]. In addition, lncRNA linc-MD1 can control muscle differentiation by serving as a‘sponge’to titrate microRNAs[7].

Increasing number of evidence demonstrated that miRNAs have oncogenic or tumor-suppressive functions[16]. Interestingly, miRNA expressions can be regulated epigenetically by EZH2 [17]. EZH2, a methyltransferase, which is a key catalytic subunit of PRC2 (EZH2, SUZ12 and EED), functions as a histone methyltransferase that specifically induces transcriptional incompetent histone H3 lysine 27 tri-methylation (H3K27me3) to the targeted genes [18]. PRC2 Complex and miRNAs are both significant mediators in carcinogenesis. Therefore, a critical issue for better understanding is that how miRNAs are specifically regulated by PRC2.

Several long non-coding RNAs have recently been reported to have a direct role in recruiting PRC2 complexes to specific loci and repress gene expression including ANRIL [19-21]. Long noncoding RNA ANRIL (CDKN2B antisense RNA 1) is transcribed as a 3.8-kb lncRNA in the opposite direction from the INK4B-ARF-INK4A gene cluster [22]. Recently, common disease genomewide association studies (GWAS) have identified ANRIL gene as a genetic susceptibility locus shared associated by coronary disease, intracranial aneurysm, type 2 diabetes and also cancers[23]. In addition, ANRIL is the best replicated genetic risk locus of coronary artery disease (CAD) and periodontitis (PD)[24]. Kyoko et al has showed that higher levels of lncRNA ANRIL expression were seen in prostate cancer and involved in repressing of the p15/CDKN2B-p16/CDKN2Ap14/ ARF gene cluster in *Cis* by directly binding to the Polycomb Repressor Complex (PRC) [22]. These results indicate that the dysregulation of ANRIL could participate in diverse human disease progression. However, the functional role and underlying mechanism of ANRIL in gastric cancer remains unclear.

In the present study, we showed that ANRIL was up-regulated in GC tissues than that in corresponding non-tumor tissues and could be served as an independent predictor for overall survival in GC. Moreover, ANRIL could regulate cell growth both in vitro and in vivo. In addition, we demonstrated that ANRIL could epigenetically silence miR-99a/miR-449a by binding to PRC2, thus regulating mTOR and CDK6/E2F1 pathway, which could in part account for ANRIL-mediated cell growth regulation. Interestingly, silencing of miR-449a by ANRIL releases E2F1 expression, and, meantime, up-regulated E2F1 promotes ANRIL expression, thus forming a positive feedback loop, continuing to promote gastric cancer cell proliferation. Our results suggest that ANRIL can crosstalk with microRNAs in the epigenetic level and facilitate the development of lncRNA-directed diagnostics and therapeutics of human gastric cancer.

## RESULTS

### Expression of *ANRIL* is up-regulated in gastric cancer tissues

**Figure 1:**
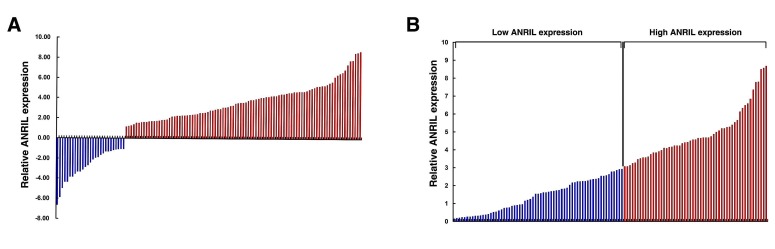
Relative *ANRIL* expression in human gastric cancer tissues (A) Relative expression of *ANRIL* in GC tissues (*N* = 120) compared with corresponding non-tumor tissues (*N* = 120). *ANRIL* expression was examined by qPCR and normalized to GAPDH expression. Results are presented as the fold-change in tumor tissues relative to normal tissues. (B) *ANRIL* expression was classified into two groups

The level of *ANRIL* was detected in 120 paired GC tissues and adjacent normal tissues by qRT-PCR, and normalized to *GAPDH*. Furthermore, *ANRIL* expression was significantly up-regulated in 77.5% (93 of 120) cancerous tissues compared with normal counterparts (P<0.01) (Figure 1A). To assess the correlation of *ANRIL* expression with clinicopathologic data, according to the relative *ANRIL* expression in tumor tissues, the 120 GC patients were classified into two groups: relative high group (n=55, fold change ≥ 3) and relative low group (n=65, fold change ≤ 3) (Figure 1B).

### Overexpression of *ANRIL* is associated with tumor size, TNM stage and poor prognosis of GC

To further understand the significance of *ANRIL* overexpression in gastric cancer, we set out to identify the potential associations between *ANRIL* expression and patients' clinicopathological features. Several clinicopathological features of 120 GC patients were summarized in Table 1. The detailed relationships between *ANRIL* expression status and clinicopathological variables of 120 patients were also shown in Table 1. Noticeably, high *ANRIL* expression in GC was significant correlation with tumor size (p = 0.001), and advanced TNM stage (p=0.041). However, *ANRIL* expression was not associated with other parameters such as age (p = 0.252) and gender (p = 0.295) et.al in GC (Table 1).

**Table 1:** Correlation between *ANRIL* expression and clinicopathological characteristics of gastric cancer

Clinical parameter	ANRIL		Chi-squared test P-value
	High No. cases (N=55)	Low No. cases N=(65)	
Age (years)			0.252
<50	23	34	
>50	32	31	
Gender			0.295
Male	21	31	
Female	34	34	
Size			0.001*
>5cm	33	20	
<5cm	22	45	
Histologic differentiation			0.133
Well	5	2	
Moderately	19	33	
Poorly	22	25	
Undifferentiated	9	5	
Invasion depth			0.121
T1	3	13	
T2	22	25	
T3	23	21	
T4	7	6	
TNM Stages			0.041*
I	6	17	
II	17	26	
III	27	18	
IV	5	4	
Lymphatic metastasis			0.587
Yes	34	37	
No	21	28	
Regional lymph nodes			0.461
PN0	19	30	
PN1	11	14	
PN2	15	14	
PN3	10	7	
Distant metastasis			0.731
Yes	5	4	
No	50	61	

**Figure 2:**
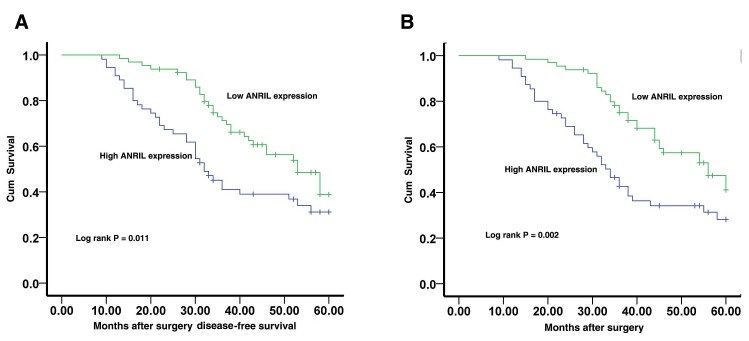
The correlation between *ANRIL* expression and the DFS or OS of gastric cancer patients Kaplan–Meier analysis of disease-free survival (A) or overall survival (B) was analyzed according to *ANRIL* expression levels.

To determine relationship between *ANRIL* expression and GC patients' prognosis, we attempted to evaluate the correlation between *ANRIL* expression and clinical outcomes. Kaplan–Meier analysis and log-rank test were used to evaluate the effects of *ANRIL* expression and the clinicopathological characteristics on disease-free survival (DFS) and overall survival (OS). The results showed that 5 years of disease-free survival (DFS) for high *ANRIL* expression is 31.1%, while is 38.8% for low *ANRIL* expression. The median survival time for high *ANRIL* expression is 32 months, while is 53 months for low *ANRIL* expression (Figure 2A, Log rank p = 0.011). Moreover, 5 years of overall survival for high *ANRIL* expression is 28.2%, while is 41.1% for low *ANRIL* expression. The median survival time for high *ANRIL* expression is 34 months, while is 56 months for low *ANRIL* expression (Figure 2B, Log rank p = 0.002).

To further assess whether *ANRIL* expression can be identified as a prognostic predictor for GC patients, the univariate and multivariate survival analyses (Cox proportional hazards regression model) were performed. Univariate analyses of clinical variables considered as potential predictors of survival are shown in Table 2. Further analysis in a multivariate Cox proportional hazards model, *ANRIL* expression, together with TNM stage and distant metastasis were strongly associated with DFS( p = 0.036, p = 0.008, p < 0.001, respectively). Meanwhile, *ANRIL* expression, TNM stage and distant metastasis were also significantly correlated with OS in our study cohort (p = 0.036, p = 0.013, p < 0.001, respectively). The results revealed that *ANRIL* expression was an independent prognostic indicator for DFS (HR=1.717, 95% CI, 1.036-2.844; P=0.036) and OS (HR=1.743, 95% CI, 1.036-2.933; P=0.036) in patients with gastric cancer (Table 2).

**Table 2:** Univariate and multivariate Cox regression analyses *ANRIL* for DFS or OS of patients in study cohort (n = 120)

Variables	DFS			OS		
	HR	95% CI	p value	HR	95% CI	p value
Univariate analysis					.	
Age(<50years vs. >50years)	1.271	0.783-2.062	0.318	1.262	0.784-2.032	0.339
Gender(male vs. female)	0.902	0.706-1.154	0.414	0.897	0.704-1.143	0.379
tumor size( >5cm vs. <5cm )	1.268	0.997-1.614	0.053	1.394	1.098-1.768	0.006*
Histologic differentiation(Well+ Moder ately vs. Poorly+ Undifferentiated)	1.241	0.767-2.008	0.379	1.304	0.811-2.097	0.273
Invasion depth(T1+T2 vs.T3+T4)	1.383	0.597-3.204	0.450	1.548	0.669-3.581	0.307
TNM stage (III + IV vs. I+II)	2.116	1.301-3.443	0.003*	2.077	1.287-3.351	0.003*
Lymphatic metastasis(No vs. Yes)	1.533	0.933-2.518	0.092	1.60	0.981-2.607	0.06
Distant metastasis(No vs. Yes)	0.499	0.335-0.744	0.001*	0.544	0.375-0.790	0.001*
Expression of ANRIL (High vs. Low)	1.836	1.134-2.974	0.013*	2.047	1.272-3.293	0.003*
Multivariate analysis						
TNM stage (III + IV vs. I+II)	1.994	1.200-3.316	0.008*	1.889	1.142-3.123	0.013*
tumor size( >5cm vs. <5cm )	-	-	-	1.230	0.957-1.581	0.106
Distant metastasis(No vs. Yes)	0.436	0.287-0.662	<0.001*	0.471	0.317-0.699	<0.001*
Expression of ANRIL (High vs. Low)	1.717	1.036-2.844	0.036*	1.743	1.036-2.933	0.036*

### Knockdown *ANRIL* inhibits gastric cancer cell proliferation *in vitro*

**Figure 3:**
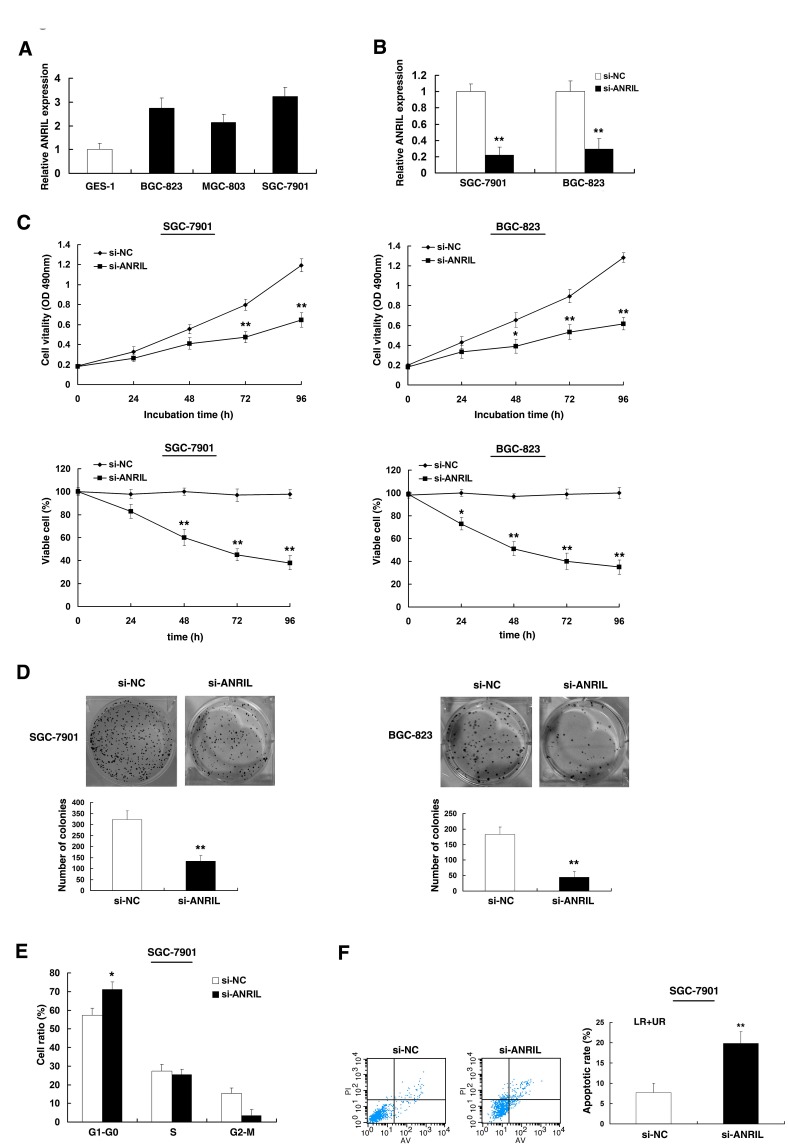
Effect of *ANRIL* on gastric cell growth *in vitro* (A) Analysis of *ANRIL* expression levels in GC cell lines (BGC-823, MGC-803, and SGC-7901) compared with the normal gastric epithelium cell line (GES-1) by qRT-PCR. (B) The relative expression level of *ANRIL* in SGC-7901 and BGC-823 cells, transfected with si-NC or si-*ANRIL*, was tested by qPCR. (C) MTT and trypan blue assays were performed to determine the cell viability and cell proliferation of SGC-7901 and BGC-823 cells. (D) Representative results of colony formation of SGC-7901 and BGC-823 cells transfected with si-NC or si-*ANRIL*. (E) At 48h after transfection, cell cycle of SGC-7901 was analyzed by flow cytometry. The bar chart represents the percentage of cells in G1–G0, S, or G2–M phase, as indicated. (F) 48 hours after transfection, SGC-7901 cells were stained and analyzed by flow cytometry. LR, early apoptotic cells. UR, terminal apoptotic cells. Error bars indicate means ± standard errors of the mean. *, P < 0.05, **, P < 0.01.

To investigate the functional role of *ANRIL* in gastric cancer cells, firstly, qRT-PCR was performed to detect the expression of *ANRIL* in diverse GC cell lines. As shown in Figure 3A, three cell lines (BGC-823, MGC-803, and SGC-7901) expressed higher levels of *ANRIL* compared with the normal gastric epithelium cell line (GES-1). Then *ANRIL* siRNA was transfected in to SGC-7901 and BGC-823 cell lines. To avoid off-target effects and ensure the efficiency of interference, we used an indeed effective interference target sequence of *ANRIL*, according to previous study [19]. qPCR assays revealed that *ANRIL* expression was significantly reduced both in SGC-7901 and BGC-823 cell lines (Figure 3B). Then MTT and trypan blue assay showed that knockdown of *ANRIL* expression significantly inhibited cell viability and cell proliferation both in SGC-7901 and BGC-823 cell lines compared with the control cells (Figure 3C). Similarly, the result of colony-formation assay revealed that clonogenic survival was significantly decreased following inhibition of *ANRIL* both in BGC-823 and SGC-7901 cell lines (Figure 3D).

Next, flow cytometric analysis was performed to further examine whether the effect of *ANRIL* on proliferation of GC cells by altering cell-cycle progression or apoptosis. The results revealed that the cell-cycle progression of SGC-7901/si-*ANRIL* cells was significantly stalled at the G1–G0 phase compared with cells transfected with si-NC (Figure 3E). In addition, knockdown *ANRIL* could obviously induce cell apoptosis (Figure 3F).

### *ANRIL* is required to target PRC2 occupancy and activity to epigenetically regulate the expression of miR-99a/miR-449a in *Trans*

**Figure 4:**
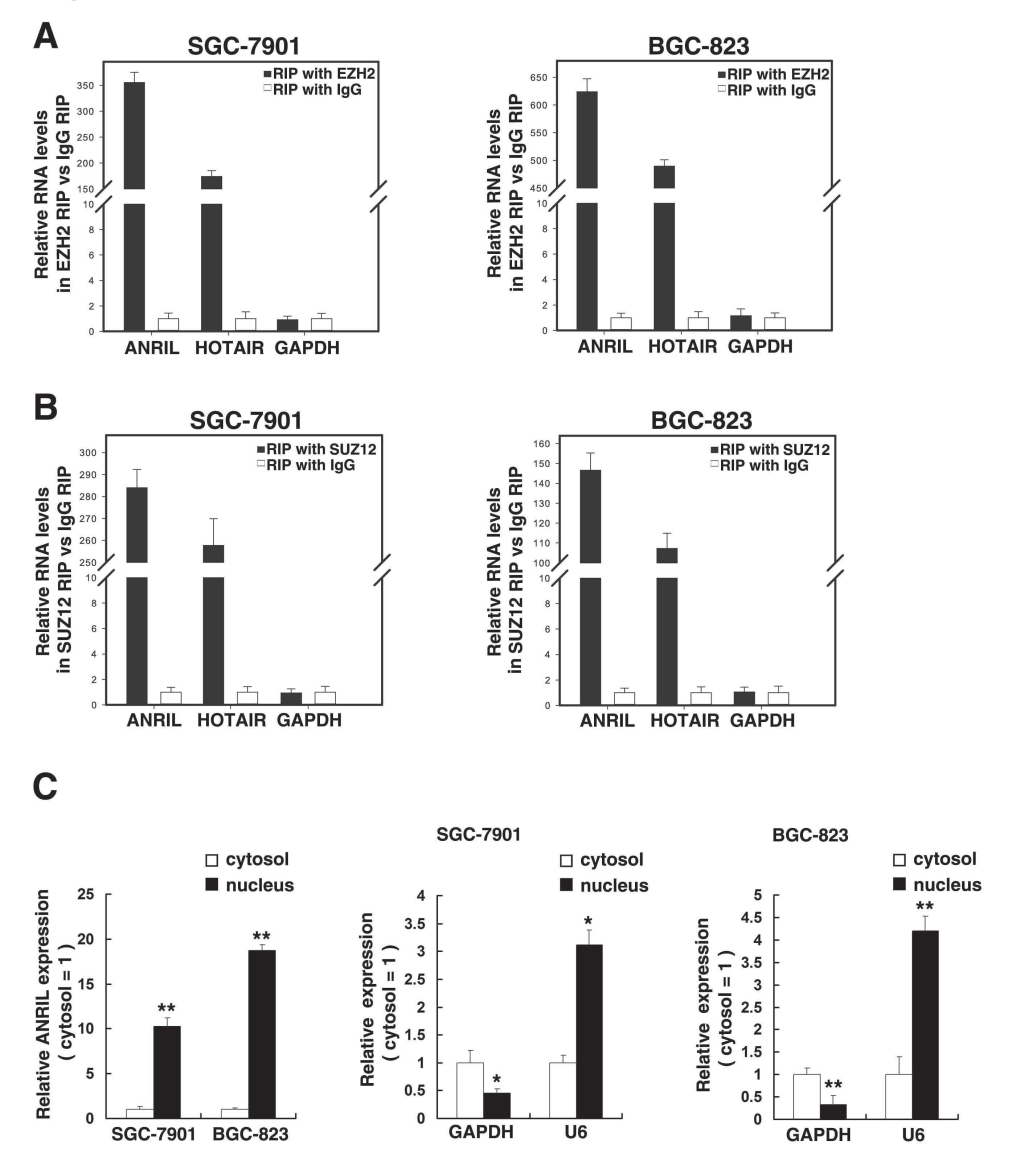
*ANRIL* could bind to PRC2, and subcellular fractionation location of *ANRIL* (A) RIP experiments were performed in SGC-7901 and BGC-823 cells and the coprecipitated RNA was subjected to qRT-PCR for *ANRIL*. HOTAIR was used as a positive control. The fold enrichment of *ANRIL* in EZH2 RIP is relative to its matching IgG control RIP. (B) The fold enrichment of *ANRIL* in SUZ12 RIP is relative to its matching IgG control RIP in SGC-7901 and BGC-823 cells. (C) *ANRIL* nuclear localization, as identified using qRT-PCR in fractionated SGC-7901 and BGC-823 cells. After nuclear and cytosolic separation, RNA expression levels in SGC-7901 and BGC-823 cells were measured by qRT-PCR. GAPDH was used as a cytosol marker and U6 was used as a nucleus marker. *, P < 0.05, **, P < 0.01.

To further study the mechanism of its regulation of gastric cancer cell proliferation, firstly, according to previous studies [19, 22], we validated that *ANRIL* whether can bind PRC2 in gastric cancer cells. As shown in Figure 4A, the endogenous *ANRIL* was enriched in the anti-EZH2 RNA immunoprecipitation (RIP) fraction relative to the input compared with the IgG fraction both in SGC-7901 and BGC-823 cell lines. Moreover, using an antibody specific to SUZ12, another member of the PRC2 complex, we also observed that endogenous *ANRIL* was obviously enriched in the anti-SUZ12 RNA-IP fraction (Figure 4B). The endogenous lncRNA HOTAIR, which binds PRC2, as determined with anti-EZH2 and anti-SUZ12 antibodies, was used as positive control [10]. In addition, we measured *ANRIL* expression in nuclear and cytosolic fractions from SGC-7901 and BGC-823 cells by qRT-PCR. The differential enrichments of GAPDH and U6 RNA were used as fractionation indicators. We found a considerable increase in *ANRIL* expression in the nucleus versus the cytosol (Figure 4C), thus suggesting that ANRIL is mainly localized in the nucleus and play a major regulatory function at the transcriptional level.

According to previous research [19, 22], *ANRIL* could epigenetically regulate p15^INK4B^ and p16^INK4A^ in *Cis* by binding to PRC2, we examined whether ANRIL could regulate p15^INK4B^ and p16^INK4A^ in gastric cancer cells. As shown in Figure S1A, p15^INK4B^ and p16^INK4A^ were induced after *ANRIL* knockdown. Moreover, by performing chromatin immunoprecipitation (ChIP), we determined that promoters of p15^INK4B^ and p16^INK4A^ were detected EZH2 binding and H3K27 trimethylation in cells transfected with si-NC. Knockdown *ANRIL* resulted in the loss of EZH2 binding and H3K27 trimethylation occupancy of p15^INK4B^ and p16^INK4A^ locus (Figure S1B). These results suggest that ANRIL promotes gastric cancer cell growth in part through epigenetic silencing of p15^INK4B^ and p16^INK4A^.

**Figure 5:**
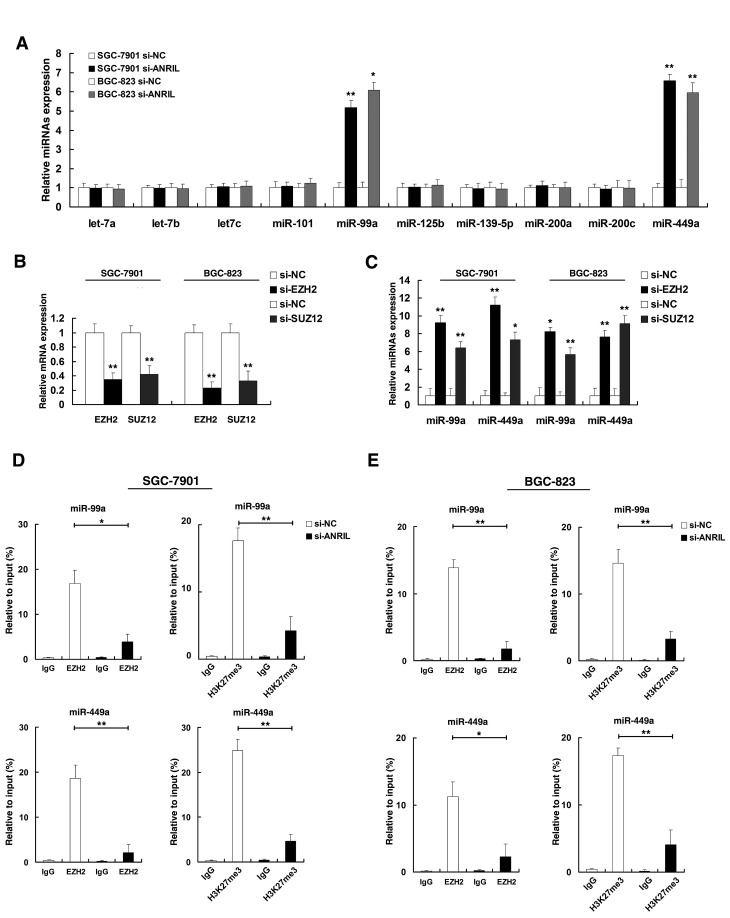
*ANRIL* is required to target PRC2 occupancy and activity to epigenetically regulate the expression of miR-99a/miR-449a in Trans (A) qRT-PCR was performed to detect miRNAs expression after *ANRIL* knockdown. (B) The relative expression level in SGC-7901 and BGC-823 cells, after knockdown EZH2 and SUZ12, was tested by qPCR. (C) The expression of miR-99a/miR-449a in SGC-7901 and BGC-823 cells, after knockdown EZH2 and SUZ12. (D) And (E) ChIP-qPCR of H3K27me3 and EZH2 of the promoter region of miR-99a/miR-449a locus after siRNA treatment targeting si-NC or si-*ANRIL* in SGC-7901 and BGC-823 cells, Antibody enrichment was quantified relative to the amount of input DNA. Antibody directed against IgG was used as a negative control.

Next, we investigated the role of *ANRIL* in epigenetic dysregulation of miRNAs, based on the analysis of the results of previous studies [17, 25], we selected 10 miRNAs. Three among them, miR-101, miR-125b and miR-139-5p have been confirmed as targets of EZH2 [17, 25]. Others were putative targets of EZH2. Then we asked whether *ANRIL* was involved in the repression of these miRNAs. As shown in Figure 5A, knockdown of *ANRIL* could significantly upregulate the expression of miR-99a/miR-449a both in SGC-7901 and BGC-823 cell lines. qPCR assays revealed that EZH2 and SUZ12 were significantly reduced both in SGC-7901 and BGC-823 cell lines (Figure 5B). The role of the PRC2 complex in coregulating suppression of these two *ANRIL*-suppressed miRNAs was investigated by EZH2 knockdown (siEZH2), and both were induced by in SGC-7901 and BGC-823 cell transfected with siEZH2 (Figure 5C). Similar results were observed for knockdown of SUZ12, another member of the PRC2 complex (Figure 5C).

Next, we determined the PRC2 complex was bound to the promoter region of miR-99a/miR-449a, and whether *ANRIL* was required for targeting PRC2 occupancy and activity of promoters of miR-99a/miR-449a. By using ChIP-qPCR assays, as shown in Figure 5D, in SGC-7901 cell line, results showed that promoters of miR-99a/miR-449a were detected EZH2 binding and H3K27 trimethylation in cells transfected with si-NC. Knockdown *ANRIL* resulted in the loss of EZH2 binding and H3K27 trimethylation occupancy of miR-99a/miR-449a locus. Similar results were also observed in BGC-823 cell line (Figure 5E). As positive controls (Figure S1C), no significant change was detected at the promoter of HOXA9, a gene regulated through EZH2 [26]. In addition, knockdown *ANRIL* could also lead to the loss of SUZ12 binding of miR-99a/miR-449a promoters (Figure S1D). These results suggested that *ANRIL* could epigenetically modulate the expression of miR-99a/miR-449a by binding to PRC2.

### *ANRIL* could indeed control target genes of miR-99a/miR-449a, thus regulating gastric cancer cell proliferation

**Figure 6:**
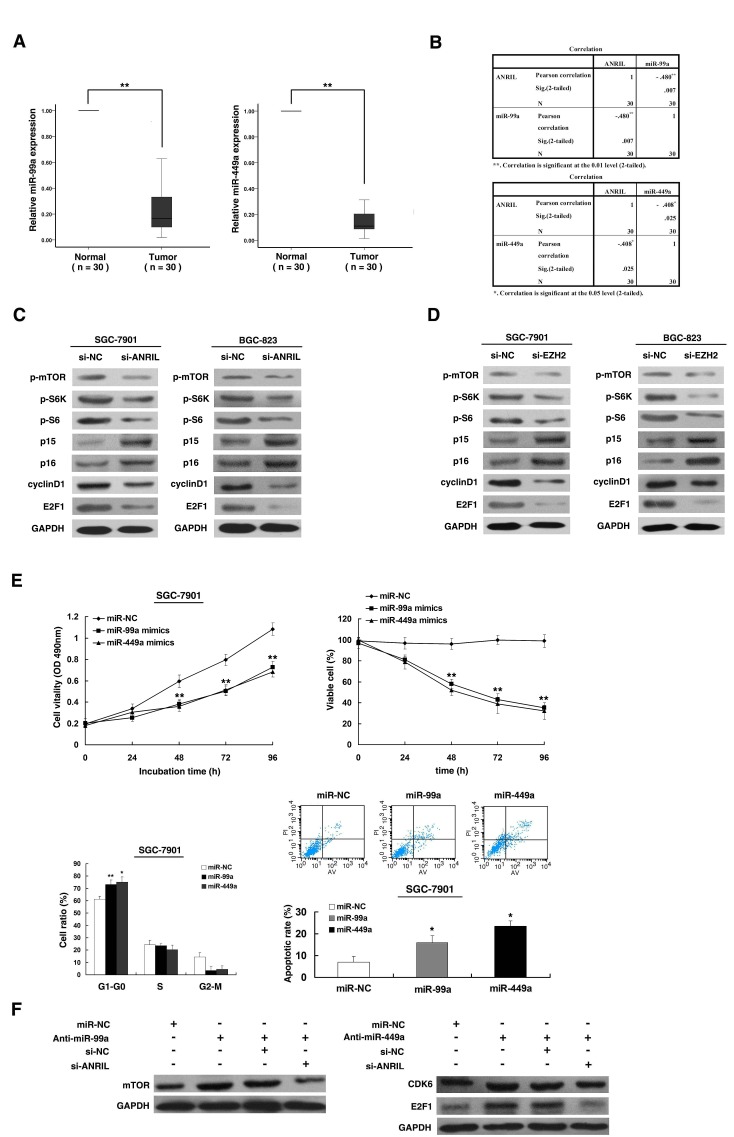
*ANRIL* could control target genes of miR-99a/miR-449a, thus regulating gastric cancer cell proliferation (A) The level of miR-99a and miR-449a was downregulated in 30 pairs GC tissues. (B) The level of miR-99a and miR-449a in GC tissues showed a statistically inverse correlation with the relative level of *ANRIL* expression (N = 30). (C) Western blot assays of p-mTOR/p-S6K/p-S6/p15/p16/cyclinD1/E2F1 after si-*ANRIL* transfection. (D) Western blot assays of p-mTOR/p-S6K/p-S6/p15/p16/cyclinD1/E2F1 after si-EZH2 transfection. (E) SGC-7901 cells were transfected with miR-99a/miR-449a mimics or miR-NC. MTT and trypan blue assays were performed to determine the cell viability and cell proliferation. Flow cytometry was performed to determine the cell cycle and apoptosis. (F) Western blot assays were performed to detect the protein level after transfection of miR-99a/miR-449a inhibitors and transfected with inhibitors followed by transfection with si-*ANRIL*.

To investigate the roles of miR-99a/miR-449a in gastric cancer, we performed qRT-PCR analysis and found that miR-99a/miR-449a expression was significantly decreased in 30 pairs of gastric cancer tissues (Figure 6A). Further analysis revealed that *ANRIL* expression was inversely correlated with miR-99a/miR-449a expression in 30 pairs of gastric cancer tissues (Figure 6B). Then we examined the well-described target genes of miR-99a/miR-449a, mTOR [27-29] and CDK6 [30-32]. To detect the impact of *ANRIL* on the activities of mTOR[33] and CDK6[34], we performed western blot assays to measure p-mTOR, phospho-S6 Kinase1, phospho-S6, p15, p16 and cyclinD1. The results revealed that the levels of p-mTOR, phospho-S6 Kinase1, phospho-S6 and cyclinD1 were decreased, and the levels of p15 and p16 were increased after *ANRIL* knockdown both in SGC-7901 and BGC-823 cell lines (Figure 6C). In addition, we also detected the important target gene of CDK6 kinases, E2F1, a pivotal role in controlling cell cycle progression[35]. Results showed that the expression of E2F1 was reduced after *ANRIL* knockdown (Figure 6C). Moreover, similar results were observed for knockdown of EZH2 (Figure 6D). Furthermore, western blot analysis showed that the expression of mTOR and CDK6 in SGC-7901 cells transfected with miR-99a/miR-449a mimics were indeed downregulated compared with cells transfected with negative control (Figure S2B). As shown in Figure S2A, SGC-7901 cells were effectively transfected with miR-99a/miR-449a mimics/ inhibitors.

To validate whether miR-99a/miR-449a could also inhibit gastric cancer cell proliferation, we enforced their expression in SGC-7901 cells with respective miRNAs mimics. Next, MTT and trypan blue assay revealed that the cells transfected with miR-99a or miR-449a had a significant growth inhibition when compared with cells transfected with miR-NC (Figure 6E). In addition, flow cytometric analysis indicated that the overexpression of miR-99a/miR-449a in SGC-7901 cells could induce obvious G1–G0 phases arrest compared with cells transfected with miR-NC and could also induce apoptosis (Figure 6E). To further confirm the regulation between *ANRIL* and miR-99a/miR-449a, we performed rescue experiments. Co-transfection (miR-99a/miR-449a inhibitors and si-*ANRIL*) could partially abrogate miR-99a/miR-449a inhibitors caused mTOR/CDK6/E2F1 stimulation (Figure 6F).

These data indicate that *ANRIL* could epigenetically modulate the expression of miR-99a/miR-449a by binding to PRC2, thus regulating mTOR and CDK6 pathway, thereby controlling gastric cancer cell proliferation.

### The released E2F1 activates *ANRIL* expression, thus forming a positive feedback loop, continuing to promote gastric cancer cell proliferation

**Figure 7:**
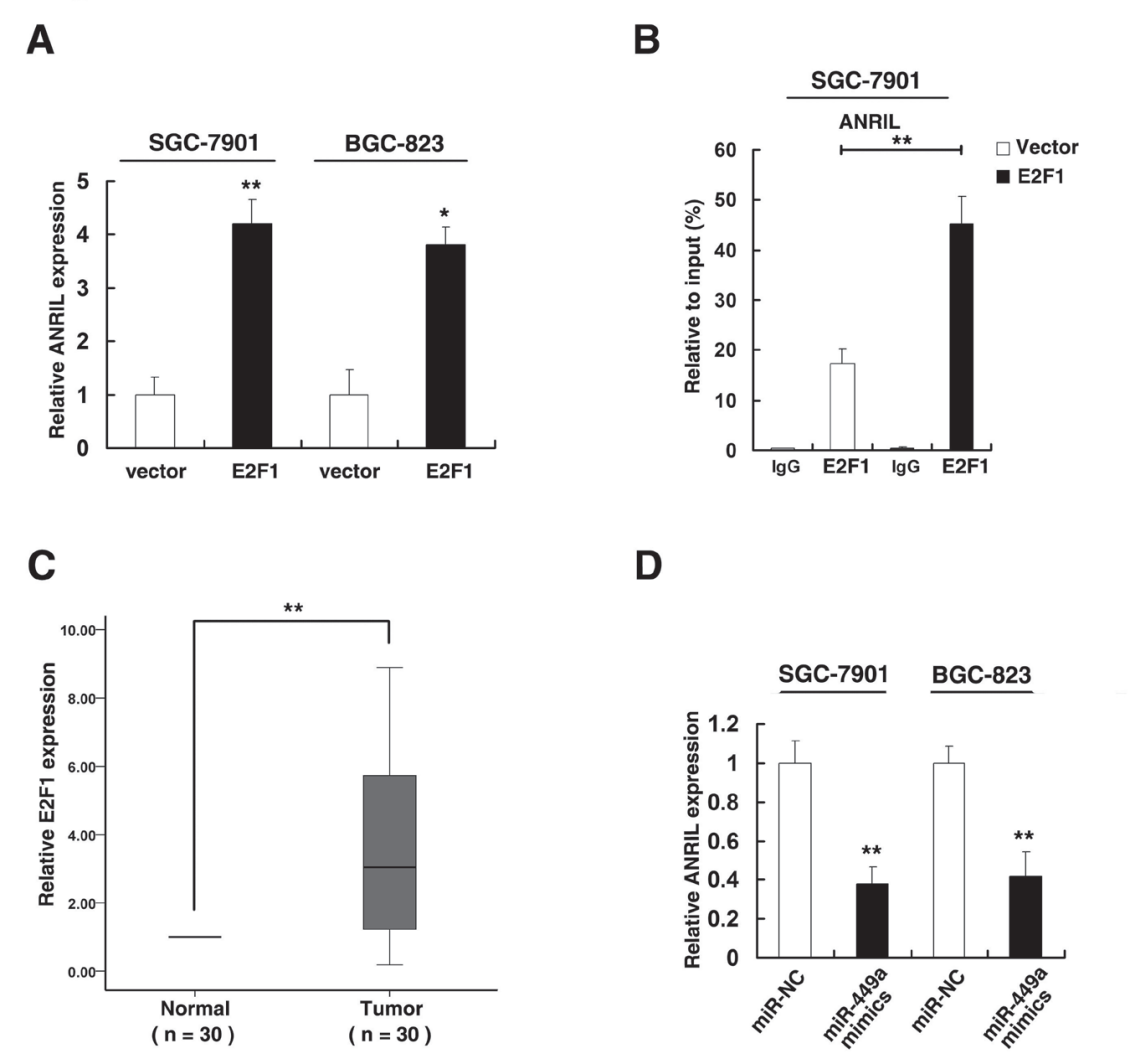
The released E2F1 activates *ANRIL* expression (A) qRT-PCR was performed to detect *ANRIL* expression after E2F1 overexpression. (B) Enrichment of E2F1 in the ANRIL promoter after E2F1 overexpression. E2F1 was immunoprecipitated and the promoter region containing E2F1-binding sequences were quantified by qRT-PCR. Control IgG immunoprecipitation was used as negative control. (C) qRT-PCR was performed to detect E2F1 expression in 30 pairs GC tissues. (D) After SGC-7901 and BGC-823 cells transfected with miR-NC or miR-449a mimics, qRT-PCR was uesd to detect *ANRIL* expression.

Our results indicated that higher expression of *ANRIL* could release E2F1 expression by silencing of miR-449a expression. According to previous study [36], E2F1 could activate *ANRIL* expression. Firstly, we enhanced E2F1 expression by transfecting E2F1 expression vector and found that the expression level of E2F1 was significantly increased in SGC-7901 and BGC-823 cell lines (Figure S2C). And enforced E2F1 expression could increase the expression of *ANRIL* (Figure 7A). ChIP assays validated that E2F1 could bind to the promoter region of *ANRIL* (Figure 7B). Moreover, qRT-PCR analysis detected the expression of E2F1 in 30 pairs gastric cancer tissues and found that E2F1 was upregulated in gastric cancer tissues (Figure 7C). Importantly, overexpression of miR-449a could suppress the expression of *ANRIL* in both SGC-7901 and BGC-823 cell lines (Figure 7D).

Collectively, higher *ANRIL* could silence the expression of p15^INK4B^, p16^INK4A^ and miR-449a. Lower expression of miR-449a releases CDK6 kinases. In addition, both p15^INK4B^ and p16^INK4A^ function as the inhibitors of CDK6[35], so lower expression of p15^INK4B^ and p16^INK4A^ also releases CDK6. The released CKD6 resulting in inactivation phosphorylation of RB, thus releasing E2F1. The released E2F1 activates *ANRIL* expression, thus forming a positive feedback loop, continuing to promote gastric cancer cell proliferation.

### Knockdown *ANRIL* inhibits gastric cancer cell proliferation *in vivo*

**Figure 8:**
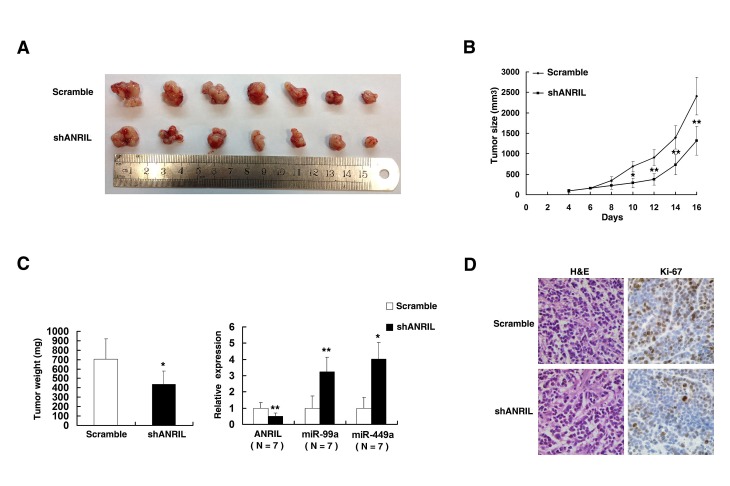
The impact of *ANRIL* on tumorigenesis *in vivo* (A) And (B) scramble or sh*ANRIL* was transfected into SGC-7901 cells, which were injected in the nude mice (n=7), respectively. Tumor volumes were calculated after injection every two days. Bars indicate SD. (C) Tumor weights are represented as means of tumor weights ±SD. qRT-PCR was performed to detect the average expression of *ANRIL*/miR-99a/miR-449a in xenograft tumors (n=7). (D) The tumor sections were under H&E staining and IHC staining using antibodies against Ki-67. Error bars indicate means ± standard errors of the mean. *, P < 0.05, **, P < 0.01.

To confirm whether the *ANRIL* affect tumorigenesis, scramble/sh*ANRIL* transfected SGC-7901 cells were inoculated into nude mice. All mice developed xenograft tumors at the injection site. As shown in Figure 8A and 8B, tumor growth in sh*ANRIL* group was significantly slower than that in the scramble vector group. Up to 16 days after injection, the average tumor weight in the sh*ANRIL* group was obviously lower than in the control group (Figure 8C). QRT-PCR analysis was conducted to detect the average expression of *ANRIL* in tumor tissues (Figure 8C). Results suggested that the average level of *ANRIL* in sh*ANRIL* group was lower than in the control group. Importantly, the average level of miR-99a/miR-449a was higher in sh*ANRIL* group (Figure 8C). We also found that the tumors developed from sh*ANRIL* cells displayed lower Ki-67 staining than that in tumors formed by scramble cells, as detected by IHC analysis (Figure 8D).

## DISCUSSION

It is becoming clear that mammalian genomes encode thousands of lncRNAs [37]. In addition to microRNAs, lncRNAs are emerging as important players in cell biology. To date, increasing evidence links dysregulation of lncRNAs to diverse human diseases including tumors[38].

In our present study, we found that the average level of *ANRIL* in GC tissues was significantly higher than those in corresponding non-tumor tissues. The high expression level of *ANRIL* in GC patients was associated with tumor size and advanced TNM stage. Moreover, high *ANRIL* expression in GC tissues was associated with a poor prognosis and could be an independent prognostic indicator. Similarly, Kyoko and colleagues have revealed that *ANRIL* was up-regulated in prostate cancer tissues [22]. In addition, aberrant expression of many other lncRNAs has also been reported to be involved in multiple tumors progression and can be used as prognostic indicators, for example enhanced *HOTAIR* expression can promote cells metastasis and serve as a prognostic factor of breast cancer[10]. *MALAT1*, a biomarker for lung cancer metastasis, can govern hallmarks of lung cancer metastasis through regulation of metastasis-related genes expression [39]. Our results indicate that *ANRIL* might function as an oncogene and exhibit important role in GC development and progression, and may be useful as a novel prognostic marker for GC.

Although *ANRIL* have been studied in a variety of physiological and pathological processes, such as glucose and fatty acid metabolism[24], neurofibromatosis type 1[40] and prostate cancer, the possible role and associated molecular mechanism of *ANRIL* in human gastric cancer remains to be clarified. Our results showed that *ANRIL* knockdown could significantly inhibit gastric cancer cell proliferation both in vitro and in vivo. In addition, flow cytometric analysis indicated that inhibitory effect of *ANRIL* on proliferation of GC cells by causing obvious G1–G0 phases arrest and inducing apoptosis.

Previous studies have showed that *ANRIL* could epigenetically regulate p15^INK4B^ and p16^INK4A^ in *Cis* by binding to PRC2 [19, 22]. The similar results were also seen in gastric cell lines (Figure S1A and S1B). However, further evidence support that most lncRNAs exercise functions in *Trans[41]*. In addition, PRC2 usually modulates a cluster of genes [10]. Thus, further experiments are needed to identify other *ANRIL*/ PRC2-regulated genes in the proliferation process of gastric cancer.

lncRNAs and microRNAs represent two classes of important non-coding RNAs in eukaryotes. Although these non-coding RNAs have been implicated in organism development and in various human diseases, little is known about the relationship between them. Recent study showed that lncRNAs could function as a ‘sponge' to titrate microRNAs[7]. Of considerable interest, epigenetic regulation machinery not only involves the regulation of mRNA[42], but also involves the regulation of multiple miRNAs[43]. In addition, deregulation of epigenetic modifiers is common in human GC[44]. EZH2 and its associated PRC2 as one of the critical epigenetic regulators also involved in the deregulation of epigenetic of miRNAs[17, 25]. Therefore, a critical issue for better understanding is that how miRNAs are specifically regulated by PRC2.

Recent studies have showed that large numbers of lncRNAs are dynamically expressed in tissue-specific patterns and have a direct role in recruiting PRC2 complexes to specific loci and repress gene expression[45]. In our study, we showed high abundance bindings between *ANRIL* and PRC2 in gastric cancer cells by using RIP, and then we asked whether *ANRIL* could mediate epigenetic regulation of miRNA, thus participating in human GC cell proliferation progression.

Previous studies determined that EZH2 epigenetically silences multiple tumor suppressor microRNAs by using microarray [17, 25]. Based on their results and analysis of the microarray data, we selected 10 miRNAs. Three among them, miR-101, miR-125b and miR-139-5p have been confirmed as targets of EZH2 by ChIP assays [17, 25]. Others were putative targets of EZH2. Then our results showed that *ANRIL* knockdown could significantly upregulate the expression of miR-99a/miR-449a both in SGC-7901 and BGC-823 cell lines in PRC2-dependent manner (Figure 5A and 5C). ChIP-qPCR assays determined that *ANRIL* is required for the PRC2 recruitment to and silencing of miR-99a/miR-449a (Figure 5D and 5E). In addition, miR-99a and miR-449a were both down-regulated in GC and further analysis revealed that expression of *ANRIL* is inversely correlated with miR-99a/miR-449a level in GC tissues (Figure 6A and 6B). Importantly, knockdown of *ANRIL* and EZH2 could control the well described target genes of miR-99a and miR-449a, mTOR [27-29] and CDK6 [30-32], also including the important target gene of CDK6 kinases, E2F1, a pivotal role in controlling cell cycle progression(Figure 6C and 6D)[35].

In an attempt to understand the biological role of miR-99a/miR-449a in GC, we enforce miR-99a/miR-449a expression by using mimics and found apparent cell proliferation inhibition through inducing obvious G1–G0 phases arrest and apoptosis (Figure 6E). These were consistent with the effects of ANRIL.

According to previous study, E2F1 could activate *ANRIL* expression [36]. As shown in Figure 7A and 7B, enforced E2F1 expression could increase the expression of *ANRIL* and E2F1 could bind to the promoter of *ANRIL*. Moreover, E2F1 was upregulated in gastric cancer tissues (Figure 7C). Higher *ANRIL* could silence the expression of miR-449a. Lower expression of miR-449a releases CDK6 kinases. Importantly, overexpression of miR-449a could significantly inhibit E2F1 expression (Figure 7D). In addition, both p15^INK4B^ and p16^INK4A^ function as the inhibitors of CDK6 [35]. And lower expression of p15^INK4B^ and p16^INK4A^ also releases CDK6. The released CKD6 resulting in inactivation phosphorylation of RB, thus releasing E2F1. The released E2F1 activates *ANRIL* expression, thus forming a positive feedback loop, continuing to promote gastric cancer cell proliferation. Besides, *ANRIL* could also silence the expression of miR-99a, thus releasing mTOR, promoting gastric cancer cell proliferation (Figure S3).

MiR-99a and miR-449a are both downregulated in a variety of tumors and indicate a poor prognosis [46, 47]. Especially, miR-449a plays an important role in regulation of cell cycle and cell proliferation [48, 49]. Similarly, mTOR and CDK6/E2F1 are both important rgulator of cell proliferation. CDK6/E2F1 plays key roles in the controlling cell cycle progression. In human cancer, the pRB-mediated repression of E2F1 is often disrupted through overactivation of cyclinD-CDK4/6 kinases [35]. In our study, higher *ANRIL* could continuously activate CDK6 through repressing miR-449a and inactivate the p15^INK4B^/p16^INK4A.^ These changes result in the inappropriate release of E2F1, consequently, gastric cancer cell proliferation.

Together, in addition through the regulation of p15^INK4B^ and p16^INK4A^ in *Cis*, *ANRIL* could also regulate the expression of miR-99a/miR-449a in *Trans,* thus controlling mTOR and CDK6/E2F1 pathway, which may in part account for *ANRIL*-mediated cell growth promotion.

To our knowledge, this is the first report showed that long noncoding RNAs could crosstalk with microRNAs in epigenetic level, thereby regulating tumor cell functions. Collectively, we showed that *ANRIL* is an important prognostic factor for GC patients and modulates GC cells proliferation both in vitro and in vivo. *ANRIL* as a member of PRC2-mediated epigenetic regulation participates in the occurrence and development of GC. Our study may supply a strategy and facilitate the development of lncRNA-directed diagnostics and therapeutics against this deadly disease.

## MATERIALS AND METHODS

### Cell culture

Three gastric cancer cell lines (SGC7901, BGC823, MGC803), and a normal gastric epithelium cell line (GES-1) were purchased from the Institute of Biochemistry and Cell Biology of the Chinese Academy of Sciences (Shanghai, China). Cells were cultured in RPMI 1640 or DMEM (GIBCO-BRL) medium supplemented with 10 % fetal bovine serum (10 % FBS), 100 U/ml penicillin, and 100 mg/ml streptomycin in humidified air at 37 °C with 5% CO2.

### Tissue samples and clinical data collection

A total of 120 patients analyzed in this study underwent resection of the primary gastric cancer at the First Affiliated Hospital of Nanjing Medical University. The study was approved by Research Ethics Committee of Nanjing Medical University (Nanjing, Jiangsu, PR China), and written informed consent was obtained from all patients. The clinicopathological characteristics of the gastric cancer patients are summarized in Table 1. Follow-up studies included physical examination, laboratory analysis, and computed tomography if necessary. Overall survival (OS) was defined as the interval between the dates of surgery and death. Disease-free survival (DFS) was defined as the interval between the dates of surgery and recurrence; if recurrence was not diagnosed, patients were censored on the date of death or the last follow-up.

### RNA extraction and qRT-PCR analyses

Total RNA was extracted from tissues or cultured cells using TRIzol reagent (Invitrogen, Carlsbad, CA). For qRT-PCR, RNA was reverse transcribed to cDNA by using a Reverse Transcription Kit (Takara, Dalian, China). Real-time PCR analyses were performed with SYBR Green (Takara, Dalian China). Results were normalized to the expression of GAPDH (for lncRNAs) or U6 (for miRNAs). Specific PCR primers for all the microRNAs and U6 were purchased from Guangzhou RiboBio Co.Ltd. The rest of primers were listed in Supplementary Table S1.

### Subcellular fractionation location

The separation of nuclear and cytosolic fractions was performed using the PARIS Kit (Life Technologies) according to the manufacturer's instructions.

### Transfection of gastric cancer cells

Gastric cancer cells were transfected with siRNA oligonucleotides, plasmids, miRNA mimics and inhibitors using Lipofectamine 2000 (Invitrogen, USA), according to the manufacturer's protocol. The nucleotide sequences of siRNA for ANRIL, EZH2 and SUZ12 were (ANRIL, GGUCAUCUCAUUGCUCUAU[19]; EZH2, GAGGUUCAGACGAGCUGAUUU[50]; SUZ12, GUCGCAACGGACCAGUUAA)[19]. Negative control siRNA (si-NC) were purchased from Invitrogen (Invitrogen, USA). The shRNA ANRIL (GGTCATCTCATTGCTCTAT) was cloned into pENTR™/U6 vector. E2F1 expression clone was purchased from Addgene. The miRNA mimics and inhibitors were purchased from Guangzhou RiboBio Co. Ltd. After transfection, cells were harvested for qRT-PCR analyses or western blot.

### Cell proliferation assays

Cell viability was tested with MTT kit (Sigma) according to the manufacturer's instruction. The antiproliferative effect was assessed by trypan blue exclusion assay (Beyotime, Haimen, China). Trypan blue staining was evaluated under the microscope and the number of viable ells (unstained blue) was counted using a hemocytometer. For colony formation assay, a certain number of transfected cells were placed in each well of 6-well plates and maintained in proper media containing 10% FBS for two weeks, during which the medium was replaced every 4 days. Colonies were then fixed with methanol and stained with 0.1% crystal violet (Sigma) in PBS for 15 minutes. Colony formation was determined by counting the number of stained colonies.

### Flow-cytometric analysis

Transfected cells were harvested after transfection by trypsinization. After the double staining with fluorescein isothiocyanate (FITC)-Annexin V and propidium iodide was done by the FITC Annexin V Apoptosis Detection Kit (BD Biosciences) according to the manufacturer's recommendations. The cells were analyzed with a flow cytometry (FACScan; BD Biosciences) equipped with a Cell Quest software (BD Biosciences). Cells were discriminated into viable cells, dead cells, early apoptotic cells, and apoptotic cells, and then the relative ratio of early apoptotic cells were compared with control transfection from each experiment. Cells for cell-cycle analysis were stained with propidium oxide by the CycleTEST PLUS DNA Reagent Kit (BD Biosciences) following the protocol and analyzed by FACScan. The percentage of the cells in G0–G1, S, and G2–M phase were counted and compared.

### Western blot assay and antibodies

Cells protein lysates were separated by 10% SDS-polyacrylamide gel electrophoresis (SDS-PAGE) transferred to 0.22μm NC membranes (Sigma) and incubated with specific antibodies. Autoradiograms were quantified by densitometry (Quantity One software; Bio-Rad). GAPDH antibody was used as control. Anti-phospho-S6 kinase1, anti-phospho-S6, anti-p15 and anti-p16 were purchased from Santa Cruz Biotechnology, Inc. Anti-phospho-mTOR, anti-mTOR, anti-CDK6, anti-cyclinD1, anti-E2F1 and anti-Ki67 were purchased from Cell Signaling Technology, Inc.

### Chromatin immunoprecipitation (ChIP) assays

ChIP assays were performed using EZ-CHIP KIT according to the manufacturer^,^ s instruction (Millipore, USA). EZH2 and SUZ12 antibodies were obtained from Abcam. E2F1 was from Cell Signaling Technology. H3 trimethyl Lys 27 antibody was from Millipore. The ChIP primer sequences were listed in Supplementary Table S1. Quantification of immunoprecipitated DNA was performed using qPCR with SYBR Green Mix (Takara). ChIP data was calculated as a percentage relative to the input DNA by the equation 2^[Input Ct- Target Ct]^ × 0.1×100.

### RNA immunoprecipitation (RIP)

RNA immunoprecipitation (RIP) experiments were performed using a Magna RIP™ RNA-Binding Protein Immunoprecipitation Kit (Millipore, USA) according to the manufacturer's instructions. Antibody for RIP assays of EZH2 and SUZ12 were from Abcam.

### Tumor formation assay in a nude mouse model

5-week-old female athymic BALB/c mice were maintained under specific pathogen-free conditions and manipulated according to protocols approved by the Shanghai Medical Experimental Animal Care Commission. SGC-7901 cells transfected with Scramble or shANRIL were harvested at a concentration of 2×10^7^ cells/ml. Of the suspending cells, 0.1 ml was subcutaneously injected into either side of the posterior flank of the nude mouse. Tumor volumes were examined every 2 days when the implantations were starting to grow bigger. Tumor volumes and weights were measured every two days in mice from the control (seven mice) or shANRIL (seven mice) groups, tumor volumes were measured (length × width^2^ × 0.5). 16 days after injection, the mice were killed and tumor weights were measured and used for further analysis. The primary tumors were excised and tumor tissues were used to perform qRT-PCR analysis of ANRIL levels and immunostaining analysis of Ki-67 protein expression.

### Immunohistochemistry (IHC)

The primary tumors were immunostained for Ki-67 as previously described [51].

### Statistical analysis

All statistical analyses were performed using SPSS 20.0 software (IBM, SPSS, USA). The significance of differences between groups was estimated by Student's t-test, χ2 test or Wilcoxon test, as appropriate. DFS and OS rates were calculated by the Kaplan-Meier method with the log-rank test applied for comparison. Survival data were evaluated using univariate and multivariate Cox proportional hazards model. Variables with a value of p<0.05 in univariate analysis were used in subsequent multivariate analysis on the basis of Cox regression analyses. Two-sided p-values were calculated, and a probability level of 0.05 was chosen for statistical significance.

## SUPPLEMENTARY FIGURES AND TABLE




