# Supplementary material for: Long noncoding RNA ANRIL indicates a poor prognosis of gastric cancer and promotes tumor growth by epigenetically silencing of miR-99a/miR-449a
Source: Oncotarget. 2014 Apr 16;5(8):2276–92. doi: 10.18632/oncotarget.1902 (PMC4039162; doi:10.18632/oncotarget.1902)
Supplement: Supplementary file 1 [file oncotarget-05-2276-s001.pdf]

Long noncoding RNA ANRIL indicates a poor prognosis of gastric cancer and promotes tumor growth by epigenetically silencing of miR-99a/miR-449a

Supplementary Material

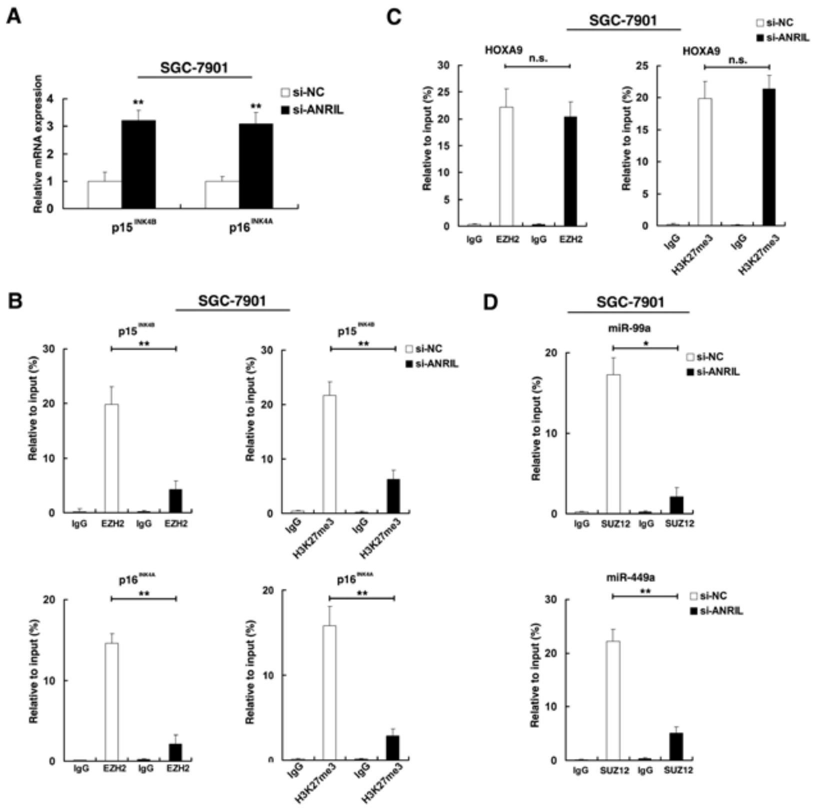

Figure S1: (A) qRT-PCR was performed to detect p15^{INK4B} and p16^{INK4A} expression after *ANRIL* knockdown. (B) ChIP-qPCR of H3K27me3 and EZH2 of the promoter region of p15^{INK4B} /p16^{INK4A} locus after siRNA treatment targeting si-NC or si-*ANRIL* in SGC-7901 cells, Antibody enrichment was quantified relative to the amount of input DNA. Antibody directed against IgG was used as a negative control. (C) ChIP-qPCR of H3K27me3 and EZH2 of the promoter region of HOXA9 after siRNA treatment targeting si-NC or si-*ANRIL* in SGC-7901 cells. Antibody enrichment was quantified relative to the amount of input DNA. (D) ChIP-qPCR of SUZ12 of the promoter region of miR-99a/miR-449a after siRNA treatment targeting si-NC or si-*ANRIL* in SGC-7901 cells, Antibody enrichment was quantified relative to the amount of input DNA.

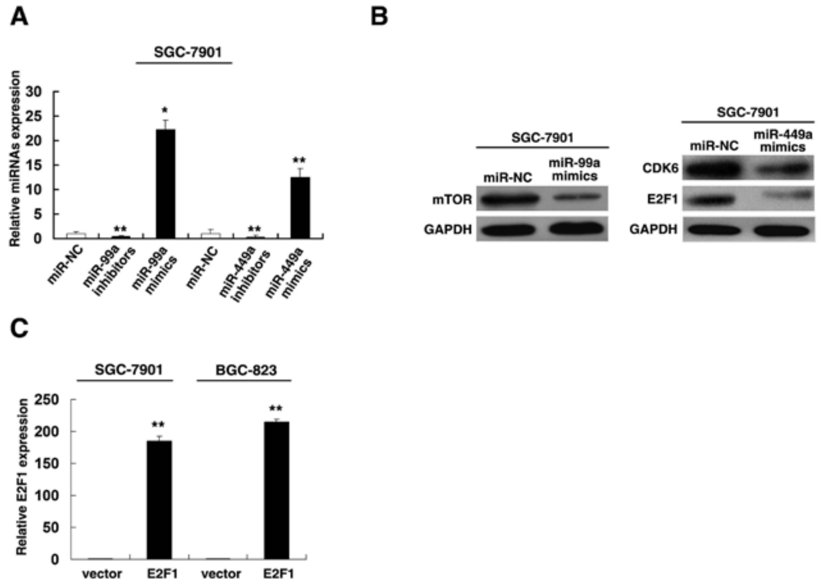

Figure S2: (A) qRT-PCR was performed to detect the expression after SGC-7901 cells transfected with miR-99a/miR-449a mimics/ inhibitors or miR-NC. (B) SGC-7901 cells were transfected with miR-99a/miR-449a mimics or miR-NC. Western blot assays of mTOR/CDK6/E2F1 after transfection. (C) qRT-PCR was uesd to detect E2F1 expression after SGC-7901/BGC-823 cells transfected with E2F1 expression plasmid or control vector.

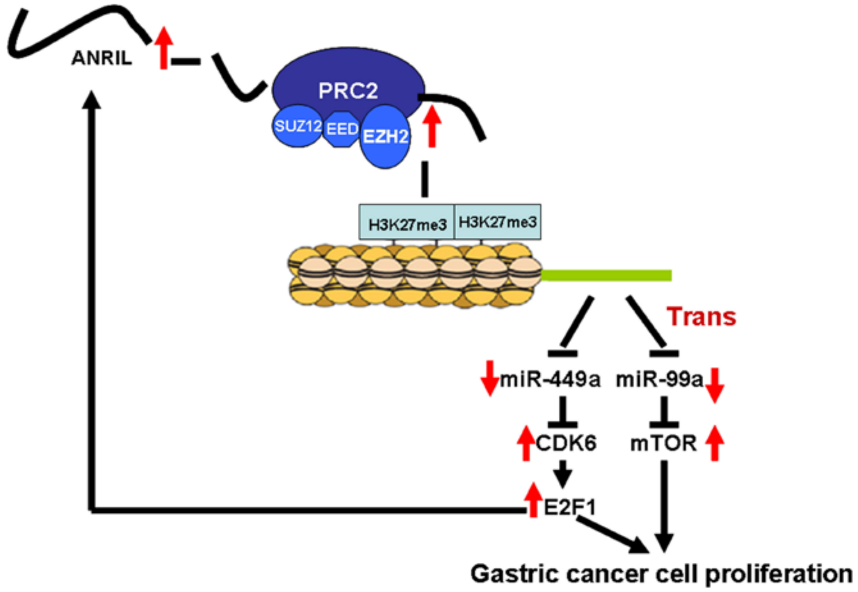

Figure S3": proposed model which medicated by *ANRIL* could form positive feedback loop in gastric cancer cell cycle progression and growth control.
